# Surgical Management of Subvalvular Aortic Stenosis in Adults: A Case Series of Two Patients

**DOI:** 10.3390/reports9010088

**Published:** 2026-03-18

**Authors:** Athanasios Papatriantafyllou, Vasileios Leivaditis, Antonella Koutela, Francesk Mulita, Spyros Papadoulas, Efstratios Koletsis, Nikolaos G. Baikoussis

**Affiliations:** 1 Department of Cardiothoracic and Vascular Surgery, Westpfalz Klinikum, 67655 Kaiserslautern, Germany; thanospap9@yahoo.gr (A.P.); vnleivaditis@gmail.com (V.L.); 2 Department of Cardiac Surgery, Ippokrateio General Hospital of Athens, 11527 Athens, Greece; antonellakout@gmail.com (A.K.); nikolaos.baikoussis@gmail.com (N.G.B.); 3 Department of General Surgery, General Hospital of Eastern Achaia—Unit of Aigio, 25100 Aigio, Greece; 4 Department of Vascular Surgery, General University Hospital of Patras, 26504 Patras, Greece; spyros.papadoulas@gmail.com; 5 Department of Cardiothoracic Surgery, General University Hospital of Patras, 26504 Patras, Greece; ekoletsis@hotmail.com

**Keywords:** subaortic stenosis, aortic valve stenosis, subvalvular aortic stenosis, subaortic membrane, aortic valve replacement, LVOT, interventricular septum hypertrophy, heart valve surgery

## Abstract

**Background and Clinical Significance:** Subvalvular aortic stenosis (SAS) is the second most common form of aortic stenosis after valvular disease and predominantly affects male patients. It is frequently associated with other congenital cardiac anomalies, such as ventricular septal defect, and is rarely diagnosed during infancy. Instead, SAS typically manifests during childhood or adulthood as a progressive left ventricular outflow tract obstruction, leading to left ventricular hypertrophy and, in many cases, aortic regurgitation. **Case Presentation:** The first patient was a 61-year-old man presenting with progressive dyspnea, in whom echocardiography revealed severe subaortic stenosis and computed tomography demonstrated aneurysmal dilatation of the ascending aorta. Intraoperatively, the aortic valve was found to be dystrophic with mixed stenotic and regurgitant disease; therefore, subaortic membrane resection, mechanical aortic valve replacement, and ascending aortic replacement with a synthetic graft were performed. The second patient was a 31-year-old man with exertional dyspnea and a discrete subaortic membrane associated with mild ascending aortic dilatation. Surgical treatment consisted of complete membrane resection and aortic valve repair, while the ascending aorta was preserved. Both patients had an uneventful postoperative course and were discharged on the fourth postoperative day. At 3-month follow-up, both were asymptomatic, in normal sinus rhythm, and demonstrated satisfactory echocardiographic findings without residual left ventricular outflow tract obstruction. **Conclusions:** Surgical intervention remains the definitive treatment for subvalvular aortic stenosis when clinically indicated. Concomitant cardiac or aortic pathology should be addressed during the same procedure to optimize outcomes. When performed with meticulous technique and appropriate patient selection, surgical correction is associated with excellent early recovery and favorable mid-term results, although long-term follow-up remains essential due to the risk of recurrence.

## 1. Introduction and Clinical Significance

Subvalvular aortic stenosis (SAS) is the second most common form of aortic stenosis and accounts for approximately 14% of left ventricular outflow tract (LVOT) obstruction [1]. Among adult congenital heart diseases, SAS represents about 6.5% of cases [2]. In the majority of patients, LVOT obstruction is caused by a discrete subaortic membrane, which may remain localized or progress to a more complex fibromuscular narrowing of the outflow tract [3,4,5].

Although traditionally considered a congenital lesion, SAS is rarely diagnosed in infancy and typically becomes clinically apparent later in life as a progressive fixed LVOT obstruction, often associated with left ventricular hypertrophy and aortic regurgitation [6,7,8]. Chronic exposure of the aortic valve to turbulent systolic flow may lead to cusp thickening, restricted mobility, and progressive valvular dysfunction, influencing both clinical presentation and surgical management [7,8,9,10].

SAS frequently coexists with other congenital cardiac abnormalities, including ventricular septal defect, coarctation of the aorta, bicuspid aortic valve, and mitral valve anomalies [3,4,5,7]. In adults, management is often individualized, depending on the extent of subvalvular obstruction and associated valvular or aortic pathology.

In this study, we present two patients with subvalvular aortic stenosis (SAS) who were successfully treated surgically in our department.

## 2. Case Presentation

### 2.1. Case 1

The first patient was a 61-year-old man who presented with symptoms consistent with aortic valve stenosis, predominantly progressive dyspnea over the preceding six months. He denied angina or syncope. His medical history was notable for overweight status and former smoking since the age of 17; no other comorbidities or previous surgical interventions were reported.

Transthoracic echocardiography revealed concentric left ventricular hypertrophy and the presence of a subaortic membrane, resulting in a mean and peak LVOT gradient of 52 and 96 mmHg, respectively, consistent with the discrete type of subvalvular aortic stenosis. Mild mitral regurgitation and mild aortic valve dysplasia with mixed mild-to-moderate regurgitation and moderate stenosis were also identified. Left ventricular systolic function was preserved, with an ejection fraction of 60%. Subsequent transesophageal echocardiography and chest computed tomography demonstrated significant dilatation of the ascending aorta, measuring 53 mm.

Given the coexistence of severe subvalvular obstruction and ascending aortic aneurysm, the patient was referred for surgical treatment. The operation was performed through a median sternotomy under cardiopulmonary bypass with cold blood cardioplegia administered via the aortic root. Custodiol^®^ cardioplegic solution was used, providing effective myocardial protection for a cross-clamp time of approximately 120 min. Arterial cannulation was achieved using an EOPA cannula placed in the aortic arch, distal to the dilated ascending aorta.

The ascending aorta was resected from the sinotubular junction to the proximal arch. The tricuspid native aortic valve, which appeared dystrophic with mixed stenotic and regurgitant disease, was excised. A discrete subaortic membrane attached to the interventricular septum was clearly identified and radically resected. A mechanical aortic valve prosthesis was implanted in the native annulus, and the ascending aorta was replaced with a 28 mm synthetic vascular graft.

### 2.2. Case 2

The second patient was a 31-year-old man who presented with exertional dyspnea. Diagnostic evaluation with transthoracic echocardiography, transesophageal echocardiography, and chest computed tomography revealed a large subaortic membrane resulting in a mean and peak LVOT gradient of 41 and 75 mmHg, respectively, and mild dilatation of the ascending aorta, measuring 41 mm, consistent with the discrete type of subvalvular aortic stenosis.

**Figure 1:**
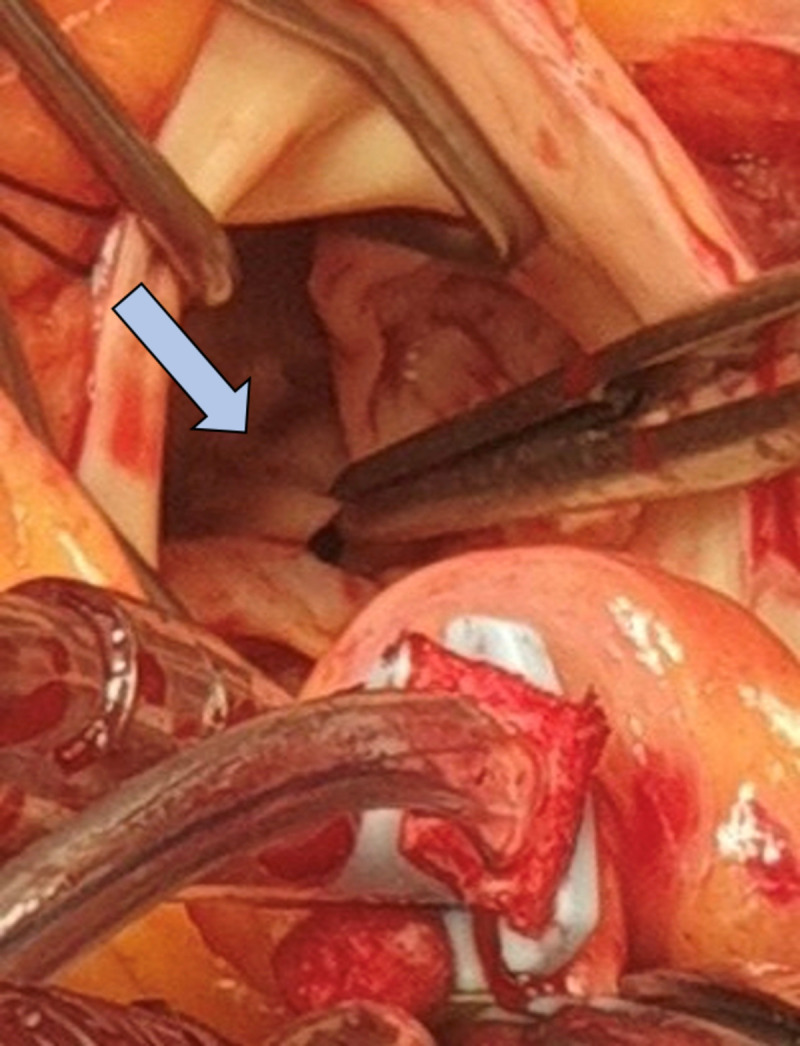
Intraoperative view demonstrating a discrete subaortic membrane located a few millimeters below the aortic valve cusps, which are gently grasped and retracted for exposure. The membrane appears as a thick, bileaflet, dystrophic fibromuscular structure situated immediately beneath the aortic annulus, causing fixed left ventricular outflow tract obstruction.

Surgical correction was performed via a minimally invasive upper mini-sternotomy. Cardiopulmonary bypass was established in a standard fashion, and cold blood cardioplegia was administered into the aortic root. An oblique aortotomy, similar to that used in aortic valve surgery, provided adequate exposure. Intraoperatively, a prominent subaortic membrane was identified immediately below the aortic valve (Figure 1). The tricuspid aortic valve was mildly thickened, with a subtle prolapse of the non-coronary cusp resulting in mild regurgitation. The subaortic membrane appeared as a thick, dystrophic, bileaflet structure located approximately 1 cm below the aortic annulus.

The membrane was completely excised through the aortic valve following gentle traction on the valve cusps. Concomitantly, the non-coronary cusp was repaired to address the mild aortic regurgitation.

### 2.3. Postoperative Course

In both patients, intraoperative transesophageal echocardiography was routinely performed to assess the adequacy of LVOT relief, aortic valve function, and mitral valve competence. This was considered mandatory for procedures involving subvalvular and valvular pathology. Custodiol^®^ cardioplegia was particularly advantageous in these complex settings, where multiple pathologies coexist.

The postoperative course was uneventful in both cases. The patients were discharged on the fourth postoperative day. Predischarge transthoracic echocardiography demonstrated satisfactory surgical results with no significant residual LVOT gradient or valvular dysfunction. At 15-day and 3-month follow-up, both patients were asymptomatic, in normal sinus rhythm, and had returned to normal daily activities.

## 3. Discussion

Subvalvular aortic stenosis (SAS) represents a heterogeneous and progressive cause of left ventricular outflow tract (LVOT) obstruction, particularly in adult patients, in whom clinical presentation and associated pathology may vary considerably [2,7]. The two cases presented herein illustrate distinct anatomical and surgical phenotypes of adult SAS and highlight the importance of individualized operative planning rather than a uniform surgical approach.

In the first patient, severe subvalvular obstruction was accompanied by advanced aortic valve degeneration and significant ascending aortic aneurysmal dilatation. This constellation reflects the long-standing hemodynamic consequences of SAS, including chronic pressure overload, turbulent systolic flow, and progressive involvement of the aortic valve and ascending aorta [2,3,7]. In such cases, isolated membrane resection would have been insufficient, as persistent valvular pathology and aortic dilatation would continue to compromise long-term outcomes. Therefore, a combined surgical strategy including membrane resection, aortic valve replacement, and ascending aortic replacement was necessary to comprehensively address all pathological components. The decision to replace the ascending aorta in the first patient was based on the presence of significant aneurysmal dilatation measuring 53 mm. Current surgical recommendations generally suggest intervention when the ascending aortic diameter exceeds 55 mm, or ≥50 mm in patients undergoing concomitant cardiac surgery.

In contrast, the second patient presented with a discrete subaortic membrane, limited aortic valve involvement, and only mild ascending aortic dilatation. This presentation is consistent with an earlier stage of disease progression, in which timely intervention may prevent irreversible valvular damage [3,4,5,7]. In this setting, valve-sparing surgery with complete membrane excision and targeted cusp repair was feasible and appropriate, allowing preservation of native valve function while effectively relieving LVOT obstruction. These differences underscore the need for careful intraoperative assessment of valve morphology and aortic dimensions to guide surgical decision-making.

Progressive aortic valve involvement is a well-recognized consequence of SAS and is primarily driven by chronic exposure to high-velocity turbulent flow directed toward the valve cusps [7,8,9,10]. This mechanism leads to cusp thickening, restricted mobility, and the development or worsening of aortic regurgitation, which may persist even after relief of the subvalvular obstruction [2,7,11]. Consequently, surgical management of SAS should not focus solely on LVOT relief but must also address associated valvular pathology to optimize long-term results.

Complete and meticulous excision of the subaortic membrane is critical, as incomplete resection has been associated with recurrence of obstruction and persistent gradients. Reported recurrence rates after surgical membrane resection range from approximately 8% to 30%, depending on patient age, anatomical characteristics, and completeness of resection. For this reason, long-term clinical and echocardiographic follow-up is recommended in adult patients following surgical correction [7,11,12]. In the present cases, particular attention was paid to radical resection of the membrane and its fibromuscular attachment to the interventricular septum in order to minimize the risk of recurrence. The risk of recurrence reflects the dynamic nature of the disease and the underlying abnormal LVOT geometry, rather than surgical failure alone [7,8]. Although both patients demonstrated excellent early outcomes without residual obstruction, lifelong echocardiographic surveillance remains essential to detect recurrence or progression of aortic regurgitation [7,13,14,15].

Ascending aortic involvement, as observed in the first case, further complicates surgical management and emphasizes the importance of comprehensive preoperative imaging [4,9,13,14]. Post-stenotic dilatation may progress independently of LVOT gradient severity and should be carefully evaluated when planning the operative strategy.

Overall, these cases highlight that adult subvalvular aortic stenosis is not a single pathological entity but rather a spectrum of disease requiring tailored surgical solutions. Early recognition, thorough imaging assessment, and individualized operative planning are essential to achieve durable relief of obstruction and favorable clinical outcomes.

The two cases presented here illustrate different stages of disease severity and anatomical involvement. While the first patient required a more extensive surgical strategy due to severe LVOT obstruction, advanced aortic valve degeneration, and significant ascending aortic dilatation, the second patient presented with a discrete membrane and limited valvular involvement, allowing successful valve-sparing surgery.

## 4. Conclusions

Subvalvular aortic stenosis is a progressive cause of left ventricular outflow tract obstruction that may present heterogeneously in adult patients. Surgical resection remains the definitive treatment when clinically indicated and should be tailored to the underlying anatomy and associated valvular or aortic pathology. The present cases illustrate the need for individualized surgical strategies and meticulous intraoperative assessment to achieve effective relief of obstruction and favorable early outcomes. Lifelong follow-up remains essential due to the risk of recurrence.

## Data Availability

The data presented this study are available from the corresponding author upon request.
